# Descartes

**Published:** 1852-10-01

**Authors:** 


### DESCARTES.

The inauguration of the statue of Descartes took place on Sunday last, the 12th of September, at Tours. A grand *cortége* was formed of the civil and military authorities, which proceeded from the Hôtel de Ville to the place where the statue had been erected, and where tribunes had been constructed for their accommodation. A great number of strangers were present, among whom was the Count de Nieuwerkerke, to whose talent the statue is due. The removal of the covering was the signal for an immense burst of acclamation from the thousands assembled.

Among the many thousand spectators who assembled to witness this august ceremony, how many cared or knew aught about the principle of the Cartesian Philosophy? There is more in posthumous fame than we had supposed;—we shall hear of statues being yet inaugurated to the memories of Plato and Aristotle. The good people of Tours wish to astonish the psychologists of Europe. Doubtless the inscription at the foot of the figure will be *non cogito ergo sum*,—an excellent text, which might lead us into a *vortex* of metaphysical disquisition!

